# *In vitro* functional characterization of prostaglandin-endoperoxide synthase 2 during chondrocyte hypertrophic differentiation

**DOI:** 10.18632/oncotarget.8889

**Published:** 2016-04-21

**Authors:** Na Li, Qian Wang, Ting Zhu, Longwei Qiao, Fei Zhang, Rui Mi, Bo Wang, Lin Chen, Junxia Gu, Yaojuan Lu, Qiping Zheng

**Affiliations:** ^1^ Department of Hematological Laboratory Science, Jiangsu Key Laboratory of Medical Science and Laboratory Medicine, School of Medicine, Jiangsu University, Zhenjiang 212013, China; ^2^ Center for Reproduction and Genetics, Suzhou Hospital Affiliated to Nanjing Medical University, Suzhou, Jiangsu, 215002, China; ^3^ State Key Laboratory of Trauma, Burns and Combined Injury, Center of Bone Metabolism and Repair, Trauma Center, Institute of Surgery Research, Daping Hospital, Third Military Medical University, Chongqing, 400042, China

**Keywords:** Cox-2, Col10a1 expression, chondrocyte hypertrophy, ATDC5, Runx2 and Alp

## Abstract

Cyclooxygenase 2 (Cox-2) has been implicated an essential role during bone repair, but the mechanisms remain elusive. Bone repair healing is known to include processes similar to endochondral ossification. In this study, we investigated the in vitro effect of Cox-2 on Col10a1 expression and chondrocyte hypertrophy, two critical components of endochondral ossification. Using quantitative RT-PCR, we detected increased mRNA levels of Cox-2 and Col10a1 in hypertrophic MCT cells, while cells treated with Cox-2 inhibitor, NS398, showed decreased mRNA and protein levels of Cox-2 and Col10a1. Increased Cox-2 also correlated with significantly upregulated Col10a1 in hypertrophic ATDC5 cells, whereas inhibition of Cox-2 significantly decreased Col10a1 expression. We further generated a Cox-2-expressing ATDC5 stable cell line. Compared with the controls, Cox-2 over-expression significantly increased Col10a1 as early as day 7 of continuous culturing, but not at days 14 and 21. Enhanced Alp staining was also observed in day 7 stable cell line. Correspondingly, we detected significantly increased levels of Runx2, Alp, Bcl-2, Bax, Col1a1, Osterix, and Bsp in day 7 stable line. Most of these genes have been associated with chondrocyte maturation and apoptosis. Together, our results support that Cox-2 promotes Col10a1 expression and chondrocyte hypertrophy in vitro, possibly through upregulation of Runx2 and other relevant transcription factors.

## INTRODUCTION

Prostaglandin-endoperoxide synthase 2 (Ptgs2) or Cox-2, is one of the family members of cyclooxygenases that catalyze biosynthesis of prostaglandins (PGs) [1, 2]. Cox-2 has been a well-known therapeutic target for the widely used non-steroidal anti-inflammatory drugs (NSAIDs) [3]. It has also been associated with multiple tumorigenesis in breast, colon, liver, and lung cancers [4–7]. More than a decade ago, Cox-2 was implicated an essential role in bone repair, as Cox-2 deficient (Cox-2^−/−^) mice showed delayed fracture healing compared with their wild type (Cox-2^+/+^) controls [8]. Bone fracture healing includes processes similar to intramembranous and endochondral ossification. Endochondral ossification typically involves cartilage intermediates that show various marker gene expression and multiple stages of chondrocyte differentiation. Notably, Cox-2 has been associated with increased expression of *Col2a1, Col10a1,* and *Sox9*, the cartilage-specific genes, during bone repair [9]. However, how Cox-2 correlates with these marker gene expression and affects chondrocyte differentiation during endochondral ossification remain unclear.

Chondrocyte hypertrophy is a critical stage of endochondral ossification characterized by expression a specific marker *Col10a1*, a gene known to play important function during skeletal development and disease [10, 11]. Cox-2 has recently been associated with chondrocyte hypertrophy, as inhibition of Cox-2 by NSAIDs impaired chondrocyte hypertrophic differentiation [12]. More recently, we detected high level of Cox-2 in hypertrophic chondrocytes and interaction between Cox-2 and *Col10a1* cis-enhancer, suggesting that Cox-2 may promote chondrocyte hypertrophy through transcriptional activation of *Col10a1* expression [13, 14].

In this study, we investigated the role of Cox-2 during endochondral ossification using two mouse chondrogenic cell models: the MCT and the ATDC5 cell lines. Both cell lines are established cell models that show significantly increased level of Col10a1 upon hypertrophic differentiation [15–17]. Here, we report the positive correlation between Cox-2 and Col10a1 expression. Cox-2 may promote chondrocyte hypertrophy through activating expression of Col10a1 and following transcription factors (TFs)/molecules, including Runx2, Alp, Bcl-2, Bax, Col1a1, Osterix, and Bsp.

## RESULTS

### Increased Cox-2 expression correlates with upregulation of Col10a1

**Figure 1:**
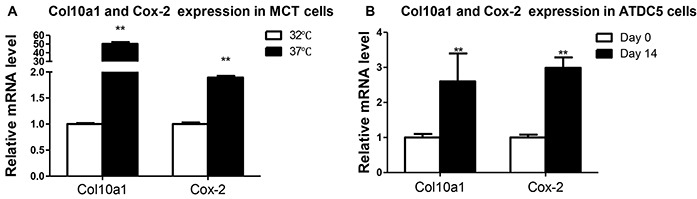
Col10a1 and Cox-2 expression in cell models of chondrocyte hypertrophy **A.** Col10a1 and Cox-2 mRNA levels were examined in proliferative (32°C) and hypertrophic (37°C) MCT cells. Col10a1 shows 50 times higher while Cox-2 mRNA level is about 2-fold in hypertrophic MCT cells compared with that of proliferative MCT cells. **B.** Both Col10a1 and Cox-2 mRNA levels were 2-3 fold upregulated in ATDC5 cells undergoing hypertrophic differentiation (day 14) compared with chondrogenic ATDC5 cells (day 0). **p<0.01.

To investigate the relationship between Cox-2 and Col10a1 expression, we have performed expression analysis of Cox-2 and Col10a1 in proliferative (growing in 32°C) and hypertrophic (growing in 37°C) MCT cells. The results showed that Col10a1 mRNA level is 50 times higher in hypertrophic than in proliferative MCT cells. Meanwhile, Cox-2 mRNA in hypertrophic MCT cells is approximately two-fold as it is in the proliferative MCT cells (Figure 1A). We also examined Cox-2 and Col10a1 mRNA transcripts in ATDC5 cells with (cultured for 14 days) or without (day 0) hypertrophic induction. The results showed that Col10a1 is approximately 3 fold upregulated in hypertrophic ATDC5 cells, while significantly increased Cox-2 (~3-fold) were also detected in hypertrophic ATDC5 cells (Figure 1B). These results suggest a positive correlation between increased Cox-2 and Col10a1 expression in hypertrophic MCT and ATDC5 cells.

### Inhibition of Cox-2 in MCT cells decreases Col10a1 expression

**Figure 2:**
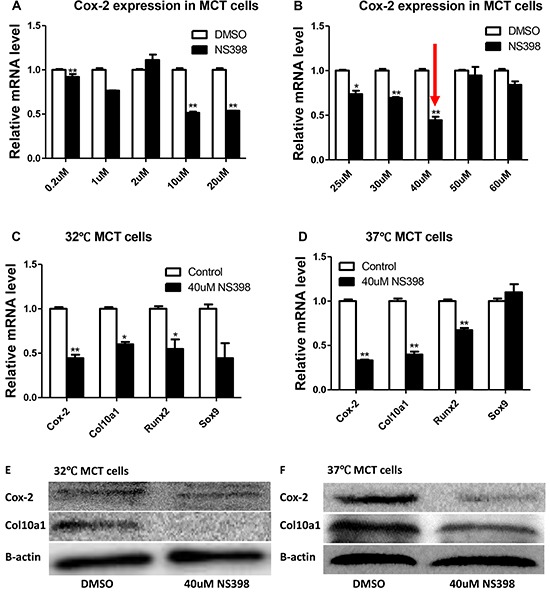
Cox-2 inhibition decreases Col10a1 expression in MCT cells Cox-2 mRNA levels showed various reduction in MCT cells treated with different concentrations of NS398 **A, B.** 40μM of NS398 showed the highest percentage of reduction. **B.** Cox-2 inhibition by NS398 (40μM) is accompanied by reduced expression of Col10a1 and Runx2, but not Sox9, both in proliferative and in hypertrophic MCT cells **C, D.** Inhibition of Cox-2 also decreased the protein levels of Col10a1 in both MCT cells as normalized to β-actin **E, F.**

To further determine the correlation between Cox-2 and Col10a1 expression, we treated MCT cells with Cox-2 inhibitor NS398 and then examined the levels of Cox-2 and Col10a1. The final concentrations of NS398 was titrated at 0.2, 1, 2, 10, 20, 25, 30, 40, 50, and 60 μM. Compared with the DMSO control, the mRNA level of Cox-2 showed the highest reduction (~60%, Figure 2A, 2B) in cells treated with 40μM of NS398. The levels of Col10a1 and Runx2 mRNAs were also decreased both in proliferative and in hypertrophic MCT cells after NS398 treatment, whereas no significant change of Sox9 mRNA was detected (Figure 2C, 2D). Correspondingly, compared with the DMSO control, the protein levels of Cox-2 and Col10a1 in both MCT cells were decreased by NS398 inhibition as detected by western blot and normalized with β-actin (Figure 2E, 2F). Together, the results suggest that MCT cells treated with NS398 resulted in decreased Cox-2 and Col10a1 expression, as well as decreased level of Runx2.

### Inhibition of Cox-2 in ATDC5 cells decreases Col10a1 expression

**Figure 3:**
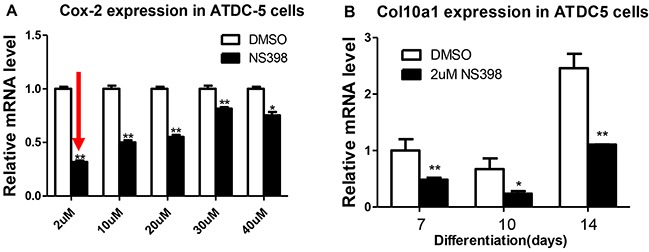
Cox-2 inhibition decreases Col10a1 expression in ATDC5 cells Compared with other concentrations, 2μM of NS398 resulted in the highest rate of reduction of Cox-2 mRNA level **A.** Inhibition Cox-2 by NS398 (2μM) decreased Col10a1 expression in ATDC5 cells undergoing chondrogenic differentiation at days 7, 10, and 14 **B.**

To confirm the correlation of Cox-2 inhibition with decreased Co10a1 expression detected in MCT cells, we have examined the levels of Cox-2 and Col10a1 in ATDC5 cells after NS398 treatment. The final concentrations of NS398 was titrated at 2, 10, 20, 30, and 40 μM. Compared with the DMSO control, Cox-2 mRNA levels showed varied reduction in all the cells treated with NS398, but 2μM of NS398 showed the highest reduction (~70%, Figure 3A). Therefore, 2μM of NS398 was selected for continuous culturing of ATDC5 cells. Col10a1 expression was measured at 7, 10, and 14 days after culturing. As expected, Col10a1 mRNA was at its highest level at day 14 after culturing compared with day 7 and 10 with or without NS398 treatment. Decreased Col10a1 expression was detected in each of the cells that were treated with NS398 compared with the DMSO controls (Figure 3B). These results suggest that inhibition of Cox-2 in ATDC5 cells decreased Col10a1 expression.

### Overexpression of Cox-2 in ATDC5 cells further increases Col10a1 expression

**Figure 4:**
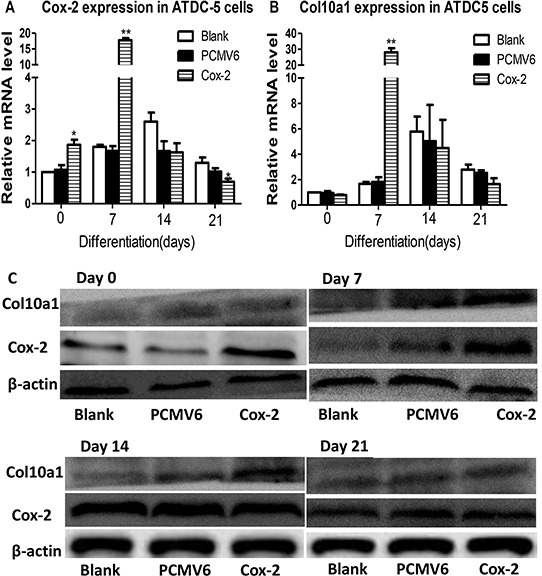
Cox-2 overexpression in ATDC5 cells upregulates Col10a1 expression **A.** The Cox-2 mRNA levels in Cox-2 stable lines cultured for 0, 7, 14, and 21 days were measured by qRT-PCR and compared with blank and vector controls. Cox-2 showed significant upregulation in the stable line compared with the vector and blank controls at day 0 and 7 with day 7 showed the highest increase of Cox-2. **B.** Significantly increased Col10a1 expression was only detected in Cox-2 expressing stable line at day 7 compared with the vector and blank controls. **C.** The protein levels of Cox-2 and Col10a1 were both significantly upregulated at day 7 in stable line compared with controls, whereas increased Cox-2 at day 0 did not increase Col10a1 expression. Despite the increased Col10a1 expression in day 14, there is no difference of Cox-2 expression between Cox-2 stable line and the controls.

We have generated a Cox-2 expressing ATDC5 stable cell line using Cox-2 expression plasmid (Origene, MR227684) with pCMV6-entry control and G418 selection. This stable cell line was subjected to continuous culturing for chondrogenic differentiation and for expression analysis. We detected the highest level of Cox-2 in cells stably transfected with Cox-2 and after cultured for 7 days compared with the blank and vector controls. Cox-2 increased at day 0, but decreased at day 21, while no significant change at day 14 (Figure 4A). We detected the highest and most significantly increased level of Col10a1 at day 7 in stable line. No significant change for Col10a1 at days 0, 14, and 21 compared with the controls (Figure 4B). We have also examined the protein levels of Cox-2 and Col10a1. The results showed that at day 0, cells transfected with Cox-2 did show significantly higher level of Cox-2 compared with the controls (Figure 4C, day 0). The protein level of Col10a1 peaks at day 7 in Cox-2 expressing stable line, which corresponds well with its mRNA level (Figure 4B, 4C, day 7). At days 14 and 21, the controls also express high levels of Cox-2, and therefore, no significant difference was detected between the stable lines and the controls. As to Col10a1, no difference was detected at days 0 and 21. It only showed moderately higher level in stable line at day 14 but significantly increased at day 7, which coincident with significant upregulation of Cox-2, compared with the controls (Figure 4C). These results demonstrated that overexpression of Cox-2 accelerates Col10a1 upregulation in ATDC5 cells as early as day 7 of culturing.

### Expression profiling of multiple marker genes in Cox-2 expressing ATDC5 cells

**Figure 5:**
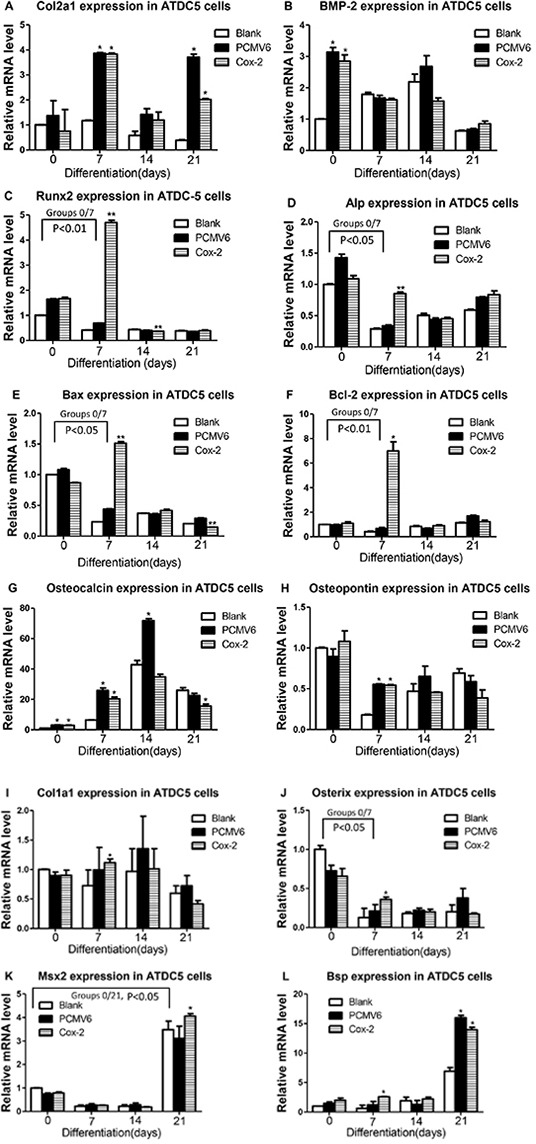
Expression analysis of marker genes in Cox-2 expressing stable line qRT-PCR was performed to examine the mRNA levels of following marker genes in Cox-2 stable line and compared with controls: No difference was shown between Cox-2 stable line and the vector control for Col2a1 **A.** and Bmp-2 **B.** in all the days examined. Runx2 **C.** and Alp **D.** were significantly increased only at day 7 in stable line compared with the vector and blank controls. Bax **E.** and Bcl-2 **F.** were significantly increased in Cox-2 stable line at day 7 compared with both controls. No difference was shown for genes Ocn **G.** and Opn **H.** between stable line and the vector control. Col1a1. **I.** Osterix **J.** and Bsp **L.** showed significant increase in stable line at day 7 compared with both controls. Msx2 **K.** showed significant increase in stable line at day 21. No difference was shown for Bsp between stable line and the vector control L. Comparison between group day 0 and each of other groups were conducted but only P values less than 0.05 or 0.01 and there is significant difference between stable line and both vector and blank controls were shown. *, p<0.05; **, p<0.01.

To investigate whether Cox-2 affects relevant marker gene expression, we have performed expression analysis of the Cox-2 expressing stable line and compared with controls. As illustrated in Figure 5, we detected significantly increased Col2a1 (A) in Cox-2 stable line at days 7 and 21 and Bmp-2 (B) at day 0 compared with blank controls. However, no obvious difference was shown between Cox-2 stable line and the pCMV6-entry vector control (A and B). Meanwhile, Runx2 (C) and Alp (D) were significantly increased only at day 7 in stable line compared with both vector and blank controls. We also detected significantly increased Bax (E), Bcl-2 (F), Ocn (G), and Opn (H) in Cox-2 stable line at day 7 compared with blank controls, but no difference was shown for genes Ocn and Opn between stable line and the vector control (G andH). Lastly, Col1a1 (I), Osterix (J), and Bsp (L) showed significant increase in stable line at day 7 compared with both controls, while Msx and Bsp also showed significant increase in stable line at day 21 but no difference was shown for Bsp between stable line and the vector control (K and L). Groups that show significant difference between stable line and both vector and blank controls were compared with group day 0 and only P values less than 0.05 or 0.01 were shown. The above genes examined have been associated with chondrocyte differentiation, maturation, apoptosis, matrix mineralization, and/or osteoblast-like differentiation. The differential expression of these marker genes suggest a potential critical function of Cox-2 during in vitro endochondral ossification in this cell model.

### Cox-2 on chondrogenic differentiation in ATDC5 cells

**Figure 6:**
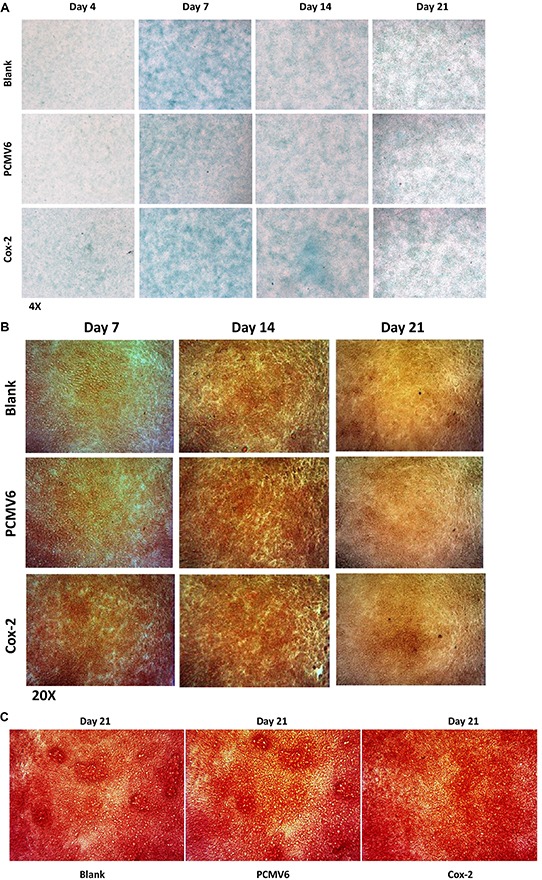
Biological effects of Cox-2 on chondrogenic ATDC5 cells **A.** The strongest Alcian blue staining was shown in cells cultured for 7 days, but no difference was observed between the stable line and the controls at all designated days of 4, 7, 14, and 21. **B.** The intensity of alkaline phosphatase (Alp) staining in cells cultured for 14 and 21 days is generally stronger than that cultured for 7 days. No obvious difference was shown for the staining intensity between Cox-2 stable lines and the controls at days 14 and 21. Much stronger Alp staining intensity was observed in Cox-2 stable line at day 7 compared with the vector and blank controls. **C.** No difference was observed for alizarin red staining between cells of Cox-2 stable line and the controls that were cultured for 21 days.

To determine the effect of Cox-2 overexpression on chondrogenic differentiation in ATDC5 cells, we have performed Alcian blue, AlP (alkaline phosphatase), and Alizarin red staining of the stable line and controls. As shown in Figure 6A, strongest Alcian blue staining indicating highest content of proteoglycans were seen in cells cultured for 7 days, but no difference was observed between the stable line and the controls. No difference was seen for the staining intensity either for days 4, 7, 14, and 21. We observed qualitatively stronger intensity of alkaline phosphatase staining in Cox-2 overexpressing cells starting at day 7 compared with the controls. Together with the increased expression of Runx2 and other marker genes, this result suggests a positive role of Cox-2 during chondrocyte maturation. However, although supportive, it is still inconclusive as to Cox-2's function during in vitro mineralization (Figure 6B). We have also performed Alizarin red staining for cells of stable line and controls cultured for 21 days, no difference was observed for the staining intensity, suggesting insignificant role of Cox-2 at late stages of in vitro ossification in this cell model (Figure 6C).

## DISCUSSION

Besides its established role in prostanoid biosynthesis, Cox-2 has been associated with the ossification processes of early bone healing[8, 19–21]. To determine its putative function during endochondral ossification, we investigated the correlation of Cox-2 with *Col10a1* expression and its effect upon chondrocyte hypertrophy. We have shown that increased Cox-2 corresponded with significantly increased level of Col10a1 in hypertrophic MCT and ATDC5 cells. We have also shown that cells treated with NS398, a selective Cox-2 inhibitor, showed decreased Col10a1 levels. In addition, overexpression of Cox-2 in ATDC5 cells further increased Col10a1 mRNA level. These results demonstrated a positive correlation between Cox-2 and Col10a1 expression. Interestingly, the highest level of Col10a1 was detected in Cox-2 stable line as early as 7 days of culturing, but not at days 14 or 21, the time when ATDC5 cells reach hypertrophic stage and show peak expression of Col10a1 [16, 22, 23].

We have examined genes Col2a1, Sox9, and Bmp-2 and no difference was detected between Cox-2 stable line and the vector control (Figure 5 and data not shown). The alcian blue staining showed no difference either. Col2a1 and Sox9 are well-known marker genes for early chondrocyte differentiation, while Bmp-2 is crucial for chondrocyte proliferation and maturation [21, 24]. Therefore, our results support an insignificant role of Cox-2 during early chondrogenesis as previously indicated [12]. Meanwhile, we detected significantly increased Alp expression along with a qualitatively stronger alkaline phosphatase staining in Cox-2 stable line cultured for 7 days. It was previously shown that Alp is expressed early in bone and calcifying cartilage and may function in the initial phases of the mineralization process [25]. Although, there is no obvious difference for alizarin red staining between stable line and the controls (data not shown), we did detect significantly increased levels of Runx2 and Osterix at day 7. Both Runx2 and Osterix are well-known essential TFs for chondrocyte maturation, osteoblast differentiation and have been shown to promote Alp expression and matrix mineralization [26–32].

Interestingly, we detected distinctly increased Bcl-2 (7 more fold) and Bax (~1.5 fold higher) in the stable lines. This differential upregulation will change the ratio of Bcl-2/Bax, supporting a potential anti-apoptotic role of Cox-2 during chondrocyte differentiation [33, 34]. Chondrocyte apoptosis is an early step of calcification of cartilage in vivo although it remains elusive as to its effect on mineralization [35, 36]. We have also detected significantly increased Col1a1 and Bsp in Cox-2 stable line at day 7. Both Col1a1 and Bsp have been shown to promote mineralization of regenerating bone during skeletal repair [37, 38]. All these results together suggest that Cox-2 upregulates Col10a1 expression and enhances chondrocyte hypertrophy, and potentially promotes matrix mineralization in vitro through upregulation of above-mentioned TFs and marker genes.

We notice that, the effect of NS398 is generally via reduction of Cox-2 enzyme activity. About 1-2 μM of NS398 may efficiently suppress Cox-2 activity which can be determined by measuring prostaglandin E2 (PGE2) in culture medium or blood plasma [12, 39]. However, NS398 may function through distinct mechanisms. It was previous shown that NS398 inhibit cell proliferation and apoptosis through transcriptional and translational regulation of its target genes P21 and P27 respectively [39]. NS398 has been shown to cause reduced Cox-2 expression indirectly via inhibition of the interaction between TFs CREB-1 and AP1s and the Cox-2 promoter [40]. The effect of NS398 may also be independent of COX-2 activity and prostaglandin synthesis, suggesting that other TFs, such as NF-kappaB and AP-1 may be required [41]. The decreased Cox-2, as well as Col10a1 expression by NS398 in our study may be attributed to altered expression of TFs, including Runx2 and Osterix [42, 43], although other molecules such as Bmp-2 may also be involved [44].

In summary, we have demonstrated a direct correlation between Cox-2 and Col10a1 expression. Cox-2 promotes Col10a1 expression and enhances hypertrophic differentiation of ATDC5 cells. Cox-2 also upregulates marker genes of chondrocyte maturation, apoptosis, and matrix mineralization, including TFs Runx2, Alp, Bsp, and Osterix etc. These findings help to explain its essential role in endochondral ossification-like changes during fracture healing, although other TFs or molecules may not be excluded.

## MATERIALS AND METHODS

### Cell lines and cell culture

The MCT cells were originally from Dr. de Crombrugghe's laboratory at MD Anderson Cancer Center (Houston, TX, USA). These large T antigen-transformed mouse chondrocytes were cultured at 32°C (proliferative) in standard DME media with 8% fetal bovine serum (FBS) and 100 U/ml penicillin and 100 μg/ml streptomycin in a humidified atmosphere with 8% CO2. These cells become hypertrophy-like cells and show significant upregulation of Col10a1 when the temperature is switched from 32°C to 37°C and continue to culture for 1, 2, or 3 days as previously described [15, 42]. The teratoma derived ATDC5 cell line were a gift from the department of orthopedic surgery at New York University Medical Center. This is an established cell model of in vitro endochondral ossification which mimic different stages of chondrogenic proliferation, hypertrophic differentiation, and bone matrix mineralization upon long and inducible culturing condition [16]. ATDC5 cells were cultured at mixed medium containing DMEM/F12 (1:1) with 5% FBS and 1% human insulin, transferrin, and sodium selenite (ITS, Sigma) at 37°C and 5% CO2 as previously described [16, 17].

### Inhibition of Cox-2 by NS398 in MCT and ATDC5 cells

MCT and ATDC5 cells were treated with Cox-2 inhibitor NS398 (S1771, Beyotime, Shanghai, China). For MCT cells, when the cells grown in 32°C reached 70-80 confluence, various concentrations (0.2, 1, 2, 10, 20, 25, 30, 40, 50, and 60 μM) of NS398 or DMSO control were added to the medium and continued to either grow in 32°C or in 37°C for 1,2, or 3 days. Total RNAs from these cells were extracted and subjected to Cox-2 expression analysis using quantitative real-time RT-PCR. The optimum concentration of NS398 that showed the highest reduction of Cox-2 mRNA was selected. For ATDC5 cells, cells were grown in 37°C until they reached 70-80 confluence before adding various concentrations (2, 10, 20, 30, 40 μM) of NS398 and DMSO control and continued to grow in 37°C for 24 hours. The optimum concentration of NS398 was also determined by expression analysis of Cox-2 mRNA in ATDC5 cells using similar strategy as that with the MCT cells. ATDC5 cells were then continually cultured for 7, 10, and 14 days with optimized concentration of NS398 and DMSO control and for further analysis.

### Transfection, establishment of Cox-2 expressing ATDC5 stable cell line

MCT cells grown in 6-well plates at 32°C and reached 70-80% confluence were used for transient transfection studies as previously described [42]. Specifically, 4 μg of Cox-2 expression plasmid (MR227684, Origene) with blank and control vector pCMV6-entry (PS100001, Origene, Rockville, MD, USA) were transfected respectively using serum-free medium and Lipofectamine-plus (GIBCO BRL). 6 hours after transfection, cells were switched to 37°C and continually cultured for 24 hours in complete medium. To establish the Cox-2 expressing stable cell line, ATDC5 cells grown in 37°C and reach 70-80% confluence were transfected with Cox-2 expressing plasmid or pCMV6-entry as a control using similar procedures as described above. Cells were then cultured in DMEM/F12 medium containing 5% FBS and neomycin G418 (600 μg/ml, 158782, MP Biomedicals). After G418 selection for 2 weeks, three colonies were picked up from the survival colonies that were confirmed to have integrated with Cox-2 expression plasmid and used for subsequent experiments.

### Total RNA isolation, RT-PCR, and quantitative real-time-PCR

Total RNAs were extracted from proliferative and hypertrophic MCT cells and ATDC5 cells using TRIzol Reagents (Invitrogen). cDNAs were reversely transcribed from 1μg of total RNA using superscript II (Invitrogen) with a total volume of 20 μl according to the manufacturer's protocols. 2μl of diluted (1:10) cDNA samples were used as template for quantitative real-time PCR (qPCR) to examine expression of following genes: Cox-2, Col10a1, Col2a1, Bmp-2, Runx2, Sox9, Alp, Bax2, Bcl-2, Osteocalcin, Osteopontin, Osterix, Col1a1, Msx2, and Bsp. The specific primers for these genes were listed in Table 1. qPCR was performed on the real-time PCR detection system from Bio-Rad using SYBR Premix Ex Taq™;II. Data were collected and analyzed by the comparative 2^−ΔΔCt^ method with Gapdh as an internal control to quantify the mRNA levels [45].

**Table 1:** Primers designed for real-time RT-PCR

Name	RefSeqID	Sense Primer (5′-3′)	Antisense Primer (5′-3′)	Amplicon (bp)
*Gapdh*	NM_008084	ACCCAGAAGACTGTGGATGG	CACATTGGGGGTAGGAACAC	171
*Runx2*	NM_001145920	ACCCAGCCACCTTTACCTAC	TATGGAGTGCTGCTGGTCTG	150
*Col10α1*	NM_009925	GCAGCATTACGACCCAAGATC	TCTGTGAGCTCCATGATTGC	201
*Sox9*	NM_011448	TTCATGAAGATGACCGACGA	ATGCACACGGGGAACTTATC	200
*Bax*	NM_007527	TGCAGAGGATGATTGCTGAC	GATCAGCTCGGGCACTTTAG	173
*Bcl-2*	NM_009741	CTGGCATCTTCTCCTTCCAG	GACGGTAGCGACGAGAGAAG	183
*Opn*	NM_009263	TGCACCCAGATCCTATAGCC	CTCCATCGTCATCATCATCG	186
*Cox-2*	NM_011198	TGCAGAATTGAAAGCCCTCT	CCCCAAAGATAGCATCTGGA	95
*Alp*	NM_007431.2	GTGAGCGCAGCCACAGAGC	GTGTGGCGTGGTTCACCCGA	134
*Msx2*	NM_013601	CCATATACGGCGCATCCTACC	CAACCGGCGTGGCATAGAG	78
*Osterix*	NM_130458	AGCGACCACTTGAGCAAACAT	GCGGCTGATTGGCTTCTTCT	121
*Osteocalcin*	NM_007541	GCAATAAGGTAGTGAACAGACTCC	GTTTGTAGGCGGTCTTCAAGC	147
*Col1a1*	NM_007742	GCAACAGTCGCTTCACCTACA	CAATGTCCAAGGGAGCCACAT	138
*Col2a1*	NM_001113515	CCTCCGTCTACTGTCCACTGA	ATTGGAGCCCTGGATGAGCA	121
*BMP-2*	NM_007553	AGAAAAGCGTCAAGCGAAACA	GTCCACGTACAAAGGGTGTCTCT	75
*Bsp*	NM_009263	AAGCAGCACCGTTGAGTATGG	CCTTGTAGTAGCTGTATTCGTCCTC	142

### Western blot

Both proliferative and hypertrophic MCT cells and ATDC5 cells under designated differentiation days were harvested, homogenized, and lysed in RIPA buffer containing proteinase inhibitor. After centrifugation, supernatant containing protein extracts were calculated and equal amount of proteins (100 μg) were used to run on SDS-PAGE gel (10 %), and then transferred onto PVDF membranes. After blocked with 5 % nonfat milk in TBS/T for 1 h, membranes were incubated with the primary antibodies anti-Cox-2 (D223097, Biotechnology, Shanghai, China) and anti-Col10a1 (sc-323750, Santa Cruz, CA, USA) at 4°C overnight. After washing, the membranes were then incubated with horseradish peroxidase–conjugated secondary antibody (goat anti-rabbit IgG antibody, D110058, Biotechnology, Shanghai, China) for 1 hour and subjected to detection using an enhanced chemiluminescence system (Minichemi, China). Anti-β-actin antibody was used in parallel as the loading control and experiments were done for three times to ensure conformance for the western assay.

### Alcian blue, ALP, and Alizarin red staining

For Alcian blue staining, ATDC5 cells from Cox-2 stable line and controls undergoing differentiation were rinsed twice with PBS, and fixed with methanol for 2 minutes at −20°C. After fixation and rinse with PBS, cells were stained overnight with 0.1% Alcian blue (A0298-1g, Biotechnology, Shanghai, China) in 0.1 N HCL, followed by wash with distilled water and observation and image analysis under Nikon microscope (Japan). For Alkaline phosphatase (ALP) staining, ATDC5 cells were stained according to manufacturer's instruction (CAKP D001-2, Jiancheng, Biotechnology Company Ltd. Nanjing, China). Briefly, cells were washed twice with PBS and fixed with 4% paraformaldehyde for 3 min, followed by incubation with freshly prepared alkaline phosphatase substrate for 15 min at 37°C in a humidified dark box. Cells were washed with PBS and counter-stained with hematoxylin-eosin before microscopic analysis. For Alizarin red staining, cells were washed twice with PBS and fixed with 95% ethanol for 10 min before staining with 1% Alizarin red (A5333, Sigma, PH 6.4) for 10 min at room temperature and then for microscopic analysis.

### Statistical analysis

Expression of marker genes by qRT-PCR was analyzed using GraphPad prism 5 software. Relative mRNA levels of marker genes and Gapdh control were quantified by the comparative 2^−ΔΔCt^ method [45]. Date were collected from three repeated runs with duplicated templates and illustrated are results of representative runs. Analysis of variance (ANOVA) was used to compare between two or more groups. P<0.05 implies significant fold changes of genes of interest in treated cells compared with controls.
